# Social and Individual Factors Associated with Eating Disorders in Young Athletes: Effects on Concentration and Fatigue

**DOI:** 10.3390/sports11070122

**Published:** 2023-06-21

**Authors:** Juan Carlos Checa Olmos, Montserrat Monserrat Hernández, Teresa Belmonte García, Diana Jiménez Rodríguez, Antonio Rodríguez Martínez, Pablo Berenguel Martínez, Antonio Miguel Berrio López

**Affiliations:** 1 Department of Geography, History and Humanities, University of Almeria, 04120 Almería, Spain; 2 Laboratory of Social and Cultural Anthropology, University of Almeria, 04120 Almería, Spain; mmh548@ual.es; 3 Department of Nursing, Physiotherapy and Medicine, University of Almeria, 04120 Almería, Spaindjr239@ual.es (D.J.R.); 4 masQsano, 04120 Almería, Spainpabloberenguelmartinez@hotmail.com (P.B.M.); 5 Junta de Andalusia, 41092 Seville, Spain

**Keywords:** eating disorders, body image, social networks, young athletes

## Abstract

Eating disorders are a growing societal problem, especially among young people. This study aims to determine the individual and social factors that support and perpetuate the risk of eating disorders (ED) and their possible consequences on an individual’s athletic and academic performances. The sample consisted of 395 athletes between 12 and 16 years of age (M = 14.07; SD = 1.35), of whom 142 (35.9%) were female, and 253 (64.1%) were male. A questionnaire was administered to collect information on sociodemographic data, body image, use of social networks, social relationships, sports practice, risk of developing ED, and academic and sports performance. In the resulting analysis, an initial cross-tabulation was carried out to observe the body distortion of the respondents as a function of BMI, followed by a linear regression to analyze the factors influencing the risk of suffering from ED. In addition, correlations were made to determine the relationship between the risk of manifesting ED and academic and sports performance. The main results show that 77.7% of the young athletes present a risk of ED (*M* = 13.3; *DT* = 3.33) due to a high body image distortion, which becomes the determining factor. In addition, relationships with family and friends have a significant influence on this. On the other hand, behaviors related with eating disorders affect concentration (r = −0.122; *p* = 0.01) and fatigue (r = −0.376; *p* < 0.01). For all these reasons, generating and promoting prevention and early detection guidelines during adolescence is necessary.

## 1. Introduction

Eating disorders (ED) constitute a public health problem [1]. These can be classified into several groups with very different characteristics [2]. Anorexia and bulimia nervosa have received the most attention in the scientific literature since they manifest similar behaviors in terms of body image, social relationships, interaction with food, etc., and whose perpetuation may be due to similar causes [3]: dissatisfaction with body image, fear of gaining weight and obsessive thoughts about food, whose perpetuation causes serious changes in the daily diet, sometimes ingesting too little and sometimes too much, accompanied by purgative behaviors [2,4,5].

Even before their manifestation and clinical detection, eating disorders affect the individual’s self-esteem, social relationships, and academic and work performances [6,7].

The scientific literature and professionals who work with the population suffering from this disorder maintain that this is a multifactorial phenomenon: biological, psychological, familiar, and social. For this reason, the socio-ecological model of analysis is particularly significant [8] and is organized around three factors: one, sociocultural, which considers the different scenarios in which the subject develops; two, sociodemographic, defined by gender or age; and three, psychological, inherent to each subject. Consequently, three aspects come into play: personal characteristics, social characteristics and how the individual reacts to them through self-evaluation and social comparison [9].

To date, ED has been primarily considered a female problem [10,11,12]; however, it is becoming increasingly prevalent among males [13,14]. Similarly, most cases are found in adolescents between 12 and 18 years of age/old (However, this disease is increasingly affecting the adult population [15]) [16], which is a critical stage in the development of body image and increases susceptibility to possible dysmorphophobic and eating behavior disorders [17,18].

Adolescents and young people who practice sports are not exempt from this situation, given that, along with the pressures and ideals of beauty manifested in the social environment and/or peer group, they also have pressures related to their maintenance of a physique in line with improving sports performance [10,19,20]. Indeed, the term anorexia athletica was coined to refer to the set of sub-clinical eating behaviors of athletes to obtain a physique as adapted as possible to the canons set by the discipline practiced [21].

Moreover, for young athletes, in addition to the two areas mentioned above (environmental and sporting), there is the virtual scenario, where they have access, on the one hand, to famous people/sportsmen and women with whom they can compare themselves and, on the other hand, to a multitude of advertising that, at times, does not correspond normally to reality or is misleading. Social networks, the need for adaptation, and the concern for a thin ideal associated with social, familiar, and professional success could explain the physiological vulnerability to present an ED [22]. Thus, the type of network use plays an essential role in both promotion and prevention.

Instagram, Facebook and YouTube, whose main contents are images, can negatively impact adolescents’ body self-perception [23], whether they are viewers or content generators. These social media networks can engender dissatisfaction among adolescents regarding their physique due to the lack of concordance between the ideal of beauty and their reality [24]. They even use filters and/or retouch images to modify their physique to publish a perfect image that can please the greatest number of people.

There are behaviors considered dangerous by specialists in the manifestation of ED [14,17,22]. Of these, caloric restriction, not resting properly, increasing the consumption of energy drinks and practicing sport beyond healthy recommendations have been explored by the academic literature from the perspective of their relationship with fatigue and tiredness [19,20,21]; however, these studies did not have EDs as a guiding thread nor did they use the young athlete population as a study sample [19].

For all these reasons, we consider it important that attention be paid in this text to the relationship between the danger of manifesting ED with both concentration and fatigue. Since the target population is in the educational stage while practicing a competitive sport, their attention and performance requirements increase [20,21].

In short, the aim of the following paper is to determine the potential risk of showing ED with young athletes in the province of Almeria (Spain), focusing on social and individual factors [13,25] and considering the different scenarios that can act as predisposing and triggering factors to manifest this phenomenon.

As a specific aim, we want to test the relationship between ED with concentration and fatigue.

The hypotheses that guide this paper are as follows: (1) Young athletes present a high probability of suffering from ED regardless of their gender. (2) The factor with the biggest impact on ED is body self-image. (3) Presenting a high probability of suffering from ED negatively correlates with concentration and rest capacity.

### 1.1. Participants

The University of Almeria Transfer project “Analysis of factors influencing the health of young athletes and design of activities in the socio-sports field for prevention in the province of Almeria: +Social Health” is the reference framework for the study, approved by the University’s bioethics committee with reference code UALBIO2022/038.

The sample was selected voluntarily from the participants of a sports injury prevention and medical examination program by *MasqSano* for sports clubs in the Province of Almería. The sample consisted of 395 athletes between 12 and 16 years of age (M = 14.07; SD = 1.35), of whom 142 (35.9%) were female, and 253 (64.1%) were male. Regarding the type of sport practiced, there were soccer (47.1%), basketball (19.2%), handball (19%), swimming (1.8%), rugby (4.1%) and volleyball (6.3%) players.

### 1.2. Instrument

The report completed by the participants, with the help of a professional, consists of several sections: sociodemographic data, data related to body image, data regarding the use of social networks, sports practice, social relationships, and questions related to academic and sports performance.

For sociodemographic variables, we took into account age, sex, weight, height, residential neighborhood and people living at home. In the section on the use of social media, we first asked about the daily and weekly time spent, type of content viewed, type of use (upload photos or videos, view photos or videos, connect with other people or all of them), and which criteria they follow in the search for friends through social media (whether or not it is based on physical appearance).

In sports practice, we asked about the type of sport and practice hours.

To obtain information about social interaction, we asked questions such as weekly time spent interacting with friends and family, levels of fun and hobbies shared with them, type of relationship, as well as the possibility of sharing hobbies.

These questions were answered using a Likert scale where “0” meant never and “4” always.

Regarding the academic and sports performance, we asked them to answer what they perceived the level of fatigue and level of concentration to be when studying subjectively, posing answers from “yes, no more than usual” to “not ever”. Additionally, objectively, we sought to know the daily sleep hours.

When recoding the participants’ body mass index (weight/height^2^), we considered their age range (12–16 years) and the considerations that this entails. Therefore, for children under 14 years of age, we considered an underweight BMI to be less than 15 kg/m^2^, normal weight BMI to be between 15 and 21 kg/m^2^, overweight BMI between 21 and 25 kg/m^2^ and obesity to be more than 25 kg/m^2^. For subjects aged between 14 and 16, BMI underweight was set at less than 16 kg/m^2^; BMI normal weight was set at between 16 and 24 kg/m^2^, BMI overweight between 24 and 27 kg/m^2^ and obesity at more than 27 kg/m^2^ [26]. Subjective data were obtained from the assertion: “I think I am fat”, adequate for our case from previous studies [27].

Healthcare personnel use various standardized measurement scales [7]: *Sick Control One Fat Food* (SCOFF) [28], *Bulimic Investigatory Test*, *Edinburgh* (BITE) [29], *Binge Eating Scale* (BES) [30], *Bulimia Test-Revised* (BULIT-R) [31], *Eating Disorder Attitude Test* (EAT-40) [32], *Eating Disorder Attitude Test-Revised* (EAT-26) [33], *Three-Factor Eating Questionnaire/Eating Inventory* (TFEQ/EI) [34], etc. Screening instruments that professionals use as screening and study tools were employed.

Although all instruments reliably measure the relationship between food and body image, in this paper, we opted to use Scoff due to its application facilities for young people, especially in terms of quickness and question formulation and comprehension. Furthermore, for its use, we were also guided by its reliability and legitimacy in different national (α = 0.97) [28,35] and international [36,37,38] contexts.

In addition, the *Sick Control One Fat Food* (SCOFF) questionnaire [28,39] was used to analyze the presence of warning signs regarding the presence of eating disorders (ED) (anorexia nervosa or bulimia nervosa). This index originally consisted of five dichotomous items (yes, no). A point is added for each “yes”, and two or more points indicate a high probability of anorexia or bulimia nervosa. In our case, because the entire questionnaire used a 0 to 5 scale format, the Scoff index ranges from 0 to 25; thus, obtaining more than 10 points means being at risk of ED, and more than 15 points signifies being at high risk.

### 1.3. Procedure

The questionnaire was carried out in a computerized and anonymous way through the Limesurvey platform. The data were collected between September and November 2022, and the participants were interviewed individually in the presence of professionals, with the prior consent of the sports clubs and/or their legal guardians/tutors.

### 1.4. Analysis

Initially, descriptive statistics were calculated to observe the prevalence of ED. Next, to study the body distortion of the respondents, a cross-tabulation was calculated between the BMI of the subjects and the subjective diagnosis that they gave themselves.

Subsequently, following the socio-ecological model [8], we performed a linear regression to discover the different factors that have a strong influence on the manifestation of ED risk. Since the number of initial variables was very high and some had a high correlation, a factor analysis was previously carried out using the maximum likelihood method with orthogonal rotation (varimax: KMO = 0.818; sig = 0.000) to reduce dimensionality and avoid multicollinearity problems.

Finally, to estimate the possible consequences of the risk of developing ED, we correlated SCOFF with variables related to hours of sleep, concentration in studies, and physical and mental fatigue. Data analysis was performed using SPSS-27 version software for Windows.

## 2. Results

### 2.1. Prevalence of ED in the Young Athletes Surveyed

**Figure 1:**
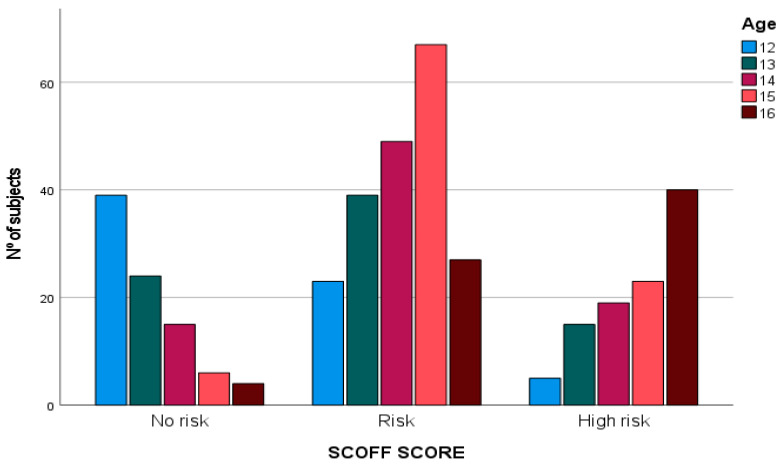
Age-related risk of developing ED. Source: Own elaboration.

According to Table 1, 77.7% of young athletes surveyed present some type of risk of developing ED and 22.3% are at high risk (M = 13.3; SD = 3.33). If we consider gender, we observe that 76.7% of women and 78.1% of men report some type of risk. Likewise, according to age (see Figure 1), it is detected that the highest risk figures for developing ED are found between 14 and 15 years old and a high risk at 16 years of age. In contrast, 12-year-olds have higher “no risk” scores.

### 2.2. Body Distortion

The mean BMI of the population was 21.3 (*SD* = 2.1). Moreover, with the BMI data grouped together, 4.1% are overweight and 0.3% obese, 84.3% have a normal weight and 11% healthy underweight. None had unhealthy underweight.

Although most of the population surveyed had “normal” BMI indices for their age group (only 4.4% were overweight or obese), 20.3% always or almost always stated, “I think I am fat”, and 30.4% sometimes did so (Table 2).

Because the data were considered disparate between the objective BMI value and its perception by the respondents, we cross-tabulated BMI and the response to the question “I think I am fat” to observe the degree of body distortion of the respondents (Table 3). The chi-square values prove that there is no relationship between “feeling fat” and BMI. Given the above, of the athletes with a BMI considered “normal”, 16.1% state that they almost always feel fat, and 3.6% always. Moreover, of the athletes with a BMI considered “underweight”, 23.3% feel fat most of the time and 2.3% always.

To find out which variables predict the variability of the risk of suffering from ED, we first reduced the different behavioral variables into factors through linear regression using the percentage of the total variance explained (TVE). The extraction reached was 66.09% (see Table 4), an acceptable result since the minimum threshold of 60% was exceeded.

Four factors were extracted (see Table 5). Factor 1, named *social and economic cooperation*, comprises the following variables: my friends and I help each other; I have enough money to do the same things as my friends; my parents give me enough money for my expenses; I can talk to my parents whenever I need to; my parents have enough money for me.

We have named Factor 2 *social networks.* It comprises the following variables: I follow someone on social networks if they are nice and make me feel good; I follow someone on social networks if they are good-looking and have a good physique; I would like to interact with more people; I would like to be the center of attention in the team.

Factor 3 takes the name of *body image* because it includes: I would like my clothes to fit better; I would like my physique to be different; I would like to be thinner.

Finally, Factor 4 is called *relationship with friends*. It comprises the following: I have spent time with my friends in the last few weeks, and I have had fun with my friends in the last week.

The linear regression was determined using the stepwise variable introduction procedure. The SCOFF index was used as the dependent variable, and the four previous factors and the individual variables: age, gender (recoded in dummy and excluded due to lack of prediction), and BMI, were used as independent variables. Table 6 shows the descriptive statistics of the variables entered.

Table 7 shows the summary of the model. It is worth noting that the R^2^ is 76%, which indicates the model’s goodness of fit and the independent variables’ high explanatory capacity for the SCOFF index’s variability.

**Figure 2:**
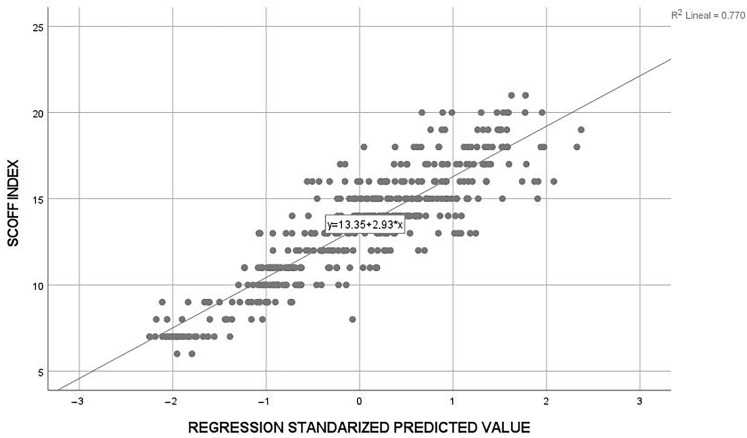
SCOFF index dispersion. Source: Own elaboration.

Similarly, taking as a reference the Durbin–Watson values with the reference values for our sample and the number of variables considered in the linear regression, it cannot be assumed that there is autocorrelation (positive or negative) (see Figure 2).

Table 8 shows the linear regression through the incorporation of different models:

In Model 1 (sig = 0.000), the variables related to body image (Factor 3) (sig < 0.001) appear with a positive sign; therefore, those who show a strong interest in how their clothes fit, would change their physique, and would like to be thinner have a higher probability of suffering from ED.

Model 2 (sig = 0.000) contains the variables related to body image (Factor 3) (sig < 0.001) and social and economic cooperation (in negative) (Factor 1) (sig < 0.001). In this case, the risk of developing ED increases when there is no intergroup peer support, lower income than their peers, or when they do not talk to their parents about their problems.

In Model 3, to the previous factors (sig < 0.000), we add age (sig < 0.001), showing that as the population studied becomes older, there is a greater risk of suffering from ED.

Finally, in Model 4 (sig < 0.001), BMI (sig = 0.002) is added, which shows that the higher this indicator is, the higher the risk of having an eating disorder is too.

In summary, it was observed that concern for body image was the most important explanatory factor for the risk of developing ED, followed by social and economic cooperation with family and friends, age and BMI. Gender and the relationship with friends’ factor were excluded from the model since they did not significantly increase the variance explained.

### 2.3. Relationship between Danger of Suffering from ED and Concentration and Rest

The final objective of the study was to observe the relationship between ED with young people concentration and rest capacity (subjective and sleeping hours). Answers to the questions posed in this regard respond to a Likert-type scale ranging from “yes, usually” to “no, never”.

Table 9 shows a significant correlation between positive SCOFF scores and poor sleep, problems with concentration, and physical and psychological fatigue. Due to this, presenting a deficient diet is related to sleeping fewer hours and having greater difficulties related to concentration, which can affect both educational and sport’s performance. Thus, eating disorders can contribute to a greater fatigue, with this variable (after training) being the one with the highest correlation. In other words, when ED occurs or is at a high risk, young athletes feel more tired after training, since his energy resources are insufficient.

## 3. Discussion

This study aimed to show which the individual and social factors that support and perpetuate the risk of eating disorders are, as well as to analyze the possible effect on adolescent athletes’ fatigue and concentration capacity.

The main findings of this study and regarding the first hypothesis show a higher prevalence of the risk of developing ED in the young population practicing sports (77.7%) compared to other studies for the same age group [16]. Moreover, the fact that the athletic population shows a higher prevalence of risk of developing ED than the non-athletic population is due to the demands or standards considered beneficial for performance [19,21], such as height [40], weight [41] and necessary meager percentage [42,43].

Additionally, unlike other studies which show greater prevalence among the female population [11,12], our study found no differences by gender since neither the descriptive data, nor the significance values in the linear regression support this. This result confirms the first hypothesis.

Therefore, given that age is a more predictive factor, we propose the need to work on prevention from this perspective rather than gender. This may be because both men and women are in a stage of constant growth and development and are constantly evaluating and comparing themselves with “others”, whether they are classmates, schoolmates, sports teams or, beyond that, celebrities followed on social networks. However, considering the results of our study, we consider it necessary and interesting for prevention to be carried out before the adolescent stage (before the age of 12 or 13).

Another aspect to highlight is the inexistence of a relationship between objective and subjective body image values. The fact that a large proportion of respondents’ report feeling fat even though BMI data show that the vast majority are normal weight [39,44] highlights two situations that support the present study’s data: (1) the presence of body distortion; and (2) body dissatisfaction. Both are related to current canons of beauty [16], in which body dissatisfaction refers to body fat [45].

Consequently, there is a well-defined line regarding the risk of ED and its relationship with body image. The result of the linear regression clearly shows that the most significant explanatory weight is given by dissatisfaction with body image, despite the normal weight found. Secondly, although they explain to a lesser degree the danger of ED, social and economic cooperative relationships become another factor explaining the pattern, to the extent that the lower level of social and economic cooperation young people have with their peers and family members, the greater the probability of suffering from eating disorders. In conclusion, these results accept the second hypothesis that guided this study.

Similarly, the data show, in our case, that social networks do not show a significant relationship with the risk of developing ED. However, the fact that it is not shown to be a determining factor in the danger of developing ED shows that it is not the consumption itself that favors ED but rather its use as a relationship and aspiration tool. That is, following someone who is good-looking, per se, does not pose a danger, but this appears when this following is used as a comparison and model to follow and/or imitate; it can create dissatisfaction with his/her body by wanting to look similarly to him/her [24,46,47].

In response to the third hypothesis that supports the relationship between the manifestation of ED and concentration and fatigue, we found a relationship between the risk of manifesting ED and feeling fatigued, getting little rest, and having problems concentrating. Other studies highlight the relationship between physiological and psychological problems with ED [2,22,48].

In our case, fatigue and the risk of developing ED are not related to the hours of sports practice, unlike previous studies where the time spent in sports practice is positively related to the risk of developing ED [45,49,50]. This particularity can be explained by the fact that, due to its competitive approach, trainers control the exercise practice of our population. However, if they engage in further sporting activity and become more tired in addition to the sporting practice prescribed by their club, they risk developing ED since this “excess” has a narcissistic rather than competitive characteristic.

## 4. Conclusions

In conclusion, we believe that this study has a high explanatory and justifying value since there is prior scientific evidence about the risk of suffering ED in young people but not specifically related to young athletes. In this way and attending to the results, we can list several proposals for action:

One: to carry out prevention programs from an early age since, as has been shown, the danger increases with the onset of adolescence. If at 13 years old, 22.9% of young athletes presents a high risk of suffering from ED, the figure rises to 56.7% when they turn 16.

Two: to implement health promotion programs where aspects of body image are also addressed in addition to working on eating and sports habits. Body image has become, also in young athletes, the greatest predictor for the risk of suffering from ED (Beta = 0.780; Sig = 0.001).

Three: design interventions based on age, in addition to gender, since the data are increasingly similar between men and women in terms of body image dissatisfaction and danger of ED. In terms of results, there are no significant differences regarding gender, but age seems to be a relevant factor (Beta = 0.108 Sig = 0.001).

Four: in the design of interventions, pay differentiated attention to the athlete and non-athlete population since, as we have observed, as in prior research [51,52], athletes show very high rates of risk of ED: 77.7% of the young athletes present a remarkable risk (*M* = 13.3; *DT* = 3.33). Even within the group of athletes, it is recommended to differentiate according to modality for diagnosis, prevention, and intervention.

Finally, five: we would like to point out, in conclusion, two issues: First, the young people participating in the study were not diagnosed, which shows that excessive concern about physical appearance can lead to behavior or attitudes that affect health before the disease is diagnosed. Moreover, if never diagnosed, these comorbidities can continuously affect lifestyle. Second, the results suggest the need for the development of longitudinal studies in which the variability of the different dimensions analyzed and their degree of influence on the risk of developing ED can be observed.

## 5. Limits

The above notwithstanding, this study presents limiting factors to be considered. The first is that the sampling is not random and therefore does not offer representative data for each group of athletes. This information is essential, especially for elite athletes, due to the demands of the different disciplines in terms of weight and/or muscle mass [21].

Second, there may be non-observational errors due to the impossibility of obtaining all the influential variables, such as psychological variables.

Third, although the surveys were short, they were administered while medical examinations were conducted, meaning the subjects’ attitudes and cooperation could be rushed or hasty.

Fourth, BMI is not a completely objective tool for diagnosing normal weight, underweight or overweight, particularly in the athletic population, since this value only considers weight and height, discarding other helpful information such as muscle mass and its ratio to fat mass [44].

Fifth, we consider age to be a limiting factor since the subjects surveyed are at a stage in which numerous changes in body composition occur due to growth and social influence.

In the absence of a longitudinal study, it is not possible to speak of a direct influence; however, this is a preliminary study with the main objective of obtaining initial data that will guide us on the needs to continue working and deepening.

Finally, sixth comprises the need for a non-athlete control group to compare the significance level of the variables in both populations.

## Figures and Tables

**Table 1:** Risk of ED in the surveyed population. Differentiation by gender and age.

SCOFF						
	Female *n* (%)	Male *n* (%)	Total *n* (%)			
No risk	33 (23.2)	55 (21.7)	88 (22.3)			
Risk	77 (54.2)	128 (50.5)	205 (51.9)			
High risk	32 (22.5)	70 (27.6)	102 (25.8)			
	AGE					
	12	13	14	15	16	Total
No risk	39 (56.9)	24 (30.8)	15 (18.1)	6 (5.3)	4 (4.5)	88
Risk	23 (35.4)	39 (50)	49 (59)	67 (70.5)	27 (38.8)	205
High risk	5 (7.7)	15 (19.2)	19 (22.9)	23 (24.2)	40 (56.7)	102

Source: Own elaboration.

**Table 2:** Response to the statement “I think I am fat”.

	N	%
Never	51	12.9
Almost never	143	36.2
Sometimes	120	30.4
Almost always	67	17
Always	13	3.3

Source: Own elaboration.

**Table 3:** Response to the statement “I think I am fat” and its relationship to BMI.

I Think I Am Fat	Body Mass Index			
	Underweight	Normal Weight	Overweight	Obesity
Never	5	45	1	0
Almost never	11	126	4	1
Sometimes	16	94	7	
Almost always	10	53	4	0
Always	1	12	0	0
Total	43	330	16	1

*X*^2^ = 9.201; *p* = 0.686. Source: Own elaboration.

**Table 4:** Total variance explained.

Component	Initial Eigenvalues			Sums of Loads Squared by Extraction		
	Total	% Variance	% Accumulated	Total	% Variance	% Accumulated
1	4.09	29.22	29.22	2.68	19.14	19.14
2	2.87	20.51	49.73	1.84	13.14	32.28
3	1.29	9.22	58.95	1.83	13.04	45.32
4	1	7.13	66.09	1.15	8.22	53.54

Extraction method: maximum likelihood. Source: Own elaboration.

**Table 5:** Degree of saturation of the items in each factor.

	Factor 1	Factor 2	Factor 3	Factor 4
I would like my clothes to fit better	−0.069	0.166	0.730	0.114
I would like a different physique	0.065	0.235	0.757	0.047
I follow someone on social networks if they are nice and make me feel good.	0.137	0.694	0.260	0.131
I follow someone on social networks if he/she is good-looking and/or has a good physique.	0.104	0.799	0.145	0.044
I would like to meet more people	0.194	0.582	0.204	0.035
I would like to be the center of attention in the team.	0.003	0.282	0.165	0.205
I would like to be thinner	−0.067	0.232	9.670	0.112
I have spent time with my friends in the last few weeks	0.253	0.118	0.163	0.790
I have had fun with my friends in the last few weeks.	0.428	0.135	0.115	0.568
My friends and I help each other				
I have enough money to do the things that my friends do.	0.569	0.084	−0.222	0.253
I have enough money to cover my expenses	0.743	0.023	−0.008	0.175
I can talk to my parents whenever I need to	0.765	−0.033	0.083	0.103
My parents have enough time for me	0.695	0.220	−0.094	0.068

Source: Own elaboration.

**Table 6:** Descriptive statistics of the variables introduced in the linear regression.

	N	Mean (SD)	Asymmetry		Kurtosis	
			Statistics	Standard Error	Statistics	Standard Error
Age	395	14.07 (1.353)	−0.95	0.123	−1.202	0.245
Body Mass Index	391	21.349 (2.199)	0.296	0.123	0.757	0.246
Factor 1	393	0.00 (0.903)	−0.065	0.123	−0.537	0.246
Factor 2	393	0.00 (0.874)	−0.066	0.123	−0.246	0.246
Factor 3	393	0.00 (0.873)	−0.061	0.123	−0.498	0.246
Factor 4	393	0.00 (0.840)	−0.373	0.123	−0.003	0.246
SCOFF Index	390	13.35 (3.334)	−0.090	0.124	−0.586	0.247

Source: Own elaboration.

**Table 7:** Model summary (dependent variable SCOFF index).

Model	Input Variables	R	R Square	Adjusted R-Squared	Durbin-Watson
1	Factor 3	0.841	0.707	0.706	
2	Factor 3	0.854	0.730	0.729	
	Factor 1				
3	Factor 3	0.862	0.751	0.749	
	Factor 1				
	Age				
4	Factor 3	0.866	0.764	0.762	1.892
	Factor 1				
	Age				
	BMI				

Source: Own elaboration.

**Table 8:** Linear regression analysis.

Variables	Model 1	Model 2	Model 3	Model 4
Body image	0.841 **	0.832 **	0.779 **	0.780 **
Social and economic cooperation with family and friends		−0.152 **	−0.139 **	−0.145 **
Age			0.128 **	0.108 **
BMI				0.081 *

** The correlation is significant at the 0.001 level. * Correlation is significant at the 0.005 level. Source: Own elaboration.

**Table 9:** Correlations between Scoff index and athletic and academic performance.

		Scoff	Hours of Sleep per Day	Do You Find it Hard to Concentrate?	Do You Feel Tired?	Do You Feel Tired in the Training Sessions?
Scoff	Pearson correlation	1	−0.251 **	−0.122 *	−0.193 **	−0.376 **
	Sig. (bilateral)		<0.001	0.01	<0.001	<0.001
	N	393	393	393	393	393

** Correlation is significant at the 0.005 level. * The correlation is significant at the 0.05 level.

## Data Availability

Not applicable.
